# Mycobacterial Osteomyelitis of the Spine Following Intravesical BCG Therapy for Bladder Cancer

**DOI:** 10.7759/cureus.545

**Published:** 2016-03-26

**Authors:** Charles E Mackel, Shane M Burke, Taylor Huhta, Ron Riesenburger, Simcha J Weller

**Affiliations:** 1 Department of Neurosurgery, Tufts University School of Medicine/Tufts Medical Center

**Keywords:** spinal osteomyelitis, mycobacterium bovis, intravesical bcg, surgical debridement, bacillus calmette-guerin

## Abstract

Osteomyelitis is an infection of the bone that can involve the vertebral column. A rare cause of vertebral osteomyelitis is *Mycobacterium bovis *after intravesical Bacillus Calmette-Guerin (BCG) therapy for transitional cell carcinoma of the bladder. In this report, we describe the case of a 64-year-old male presenting with constitutional symptoms, progressive thoracic kyphosis, and intractable T11 and T12 radiculopathies over the proceeding six months. A CT scan revealed erosive, lytic changes of the T12 and L1 vertebrae with compression of the T12 vertebra. An MRI demonstrated T11-12 osteomyelitis with intervening discitis and extensive paraspinal enhancement with a corresponding hyperintensity on a short tau inversion recovery (STIR) sequence. A needle aspiration grew out *Mycobacterial tuberculosis *complex that was pansensitive to all antimicrobial agent therapies, except pyrazinamide on culture, a finding consistent with an *M. bovis *infection. The patient’s infection and neurologic compromise resolved after transthoracic T11-12 vertebrectomies with decompression of the spinal cord and nerve roots as well as T10-L1 instrumented fusion and protracted antimicrobial therapy. The epidemiology and natural history of *M. bovis *osteomyelitis are reviewed and the authors emphasize a mechanism of vertebral inoculation to explain the predilection of *M. bovis* osteomyelitis in males after intravesical BCG therapy.

## Introduction

The incidence of vertebral osteomyelitis is currently estimated to be about 2.5/100,000, but this figure continues to increase [1]. Vertebral osteomyelitis is foremost a bacterial infection. The most common pyogenic pathogens include *Staphylococcus aureus*, *Escherichia coli*, and coagulase-negative *Staphylococcus *species. However, other etiologies have also been reported [2].

In developed countries, tuberculosis may be responsible for 9-46% of vertebral osteomyelitis [2]. Also known as Pott’s disease, tuberculous osteomyelitis typically affects the intervertebral disc space and adjoining vertebrae within the thoracolumbar region [3]. Although the predominant agent of Pott’s disease is *Mycobacterium tuberculosis*, the zoonotic pathogen, *Mycobacterium bovis,* can also infect humans, resulting in both pulmonary and vertebral disease. Current estimates suggest that *M. bovis *accounts for 1-2% of all TB cases reported in developed countries [4].

A live-attenuated strain of *M. bovis*, Bacillus Calmette–Guérin (BCG), was initially developed as a vaccine for the human form of TB but has proven to be a safe and effective therapy for various carcinomas *in situ*. Since 1976, BCG has been administered intravesically to treat transitional cell carcinoma of the bladder [5]. It is believed that the BCG mycobacteria adhere to the bladder wall and stimulate a Th1-mediated immune response, which diminishes tumor burden, by releasing interferons and recruiting natural killer cells [6]. Less than 5% of the patients treated with this technique experience a drug-related adverse event during therapy [7]. The most frequent side effects are local, namely cystitis and bladder contractures [8]. Injury to the bladder endothelium during or after treatment appears to be a prerequisite for disseminated morbidities [8]. Vertebral osteomyelitis is a rare – but serious – potential complication of intravesical BCG instillation [5]. Herein, we describe a particularly severe episode of BCG-induced tuberculous osteomyelitis. Informed patient consent was obtained for his treatment.

## Case presentation

A 64-year-old male with a medical history of transitional cell carcinoma of the bladder treated with intravesical BCG over two months presented with a six-month history of severe, progressive mid-back and chest wall pain. He had become non-ambulatory and also began to display constitutional symptoms, including night sweats and weight loss. For his oncologic care, he had undergone two transurethral resections six and 11 months prior to presentation, which had been interspaced by five instillations of intravesical BCG.

**Figure 1:**
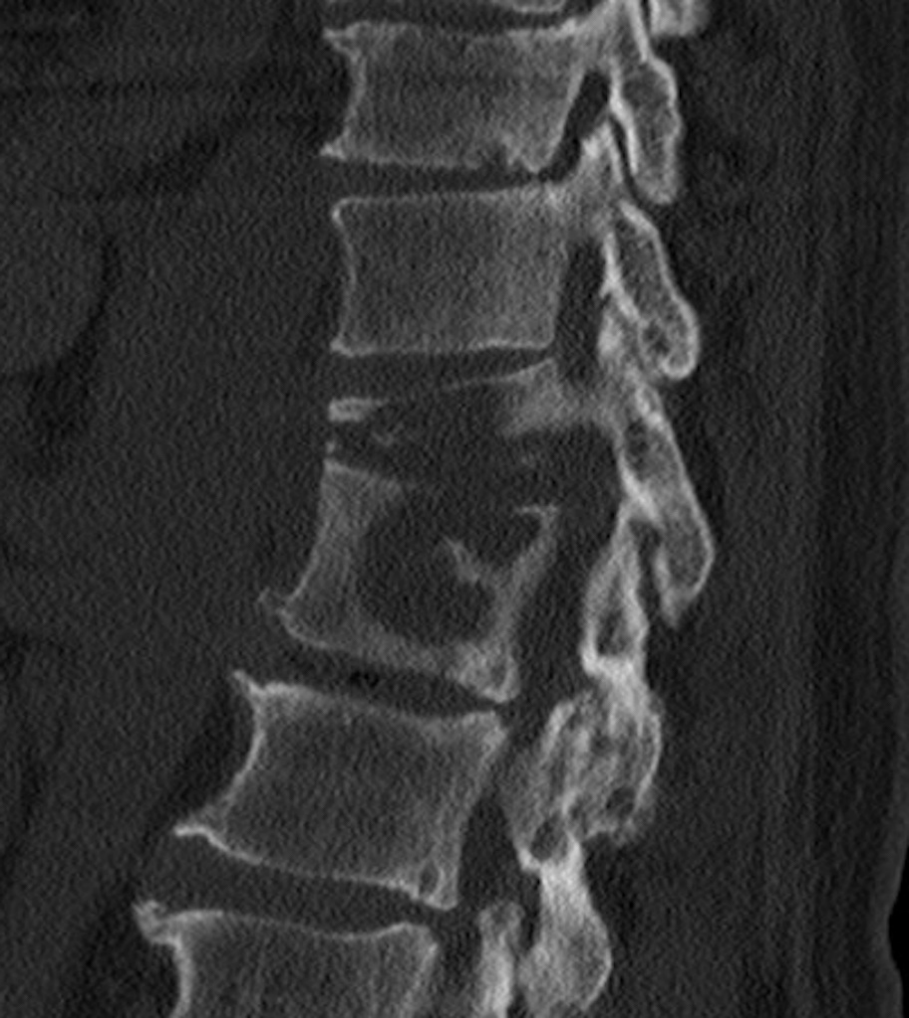
Preoperative sagittal CT Preoperative sagittal CT demonstrating erosive, lytic changes of the T11 and T12 vertebrae with compression deformity of the T11 vertebra.

The patient first presented to an outside hospital with complaints of mid-back pain six months prior to presentation. Laboratory data was notable for a moderately elevated white blood cell count of 22.1 K/μL, a C-reactive protein of 40.42 mg/L, and an erythrocyte sedimentation rate of 23 mm/hr. A chest x-ray revealed multiple, nonspecific lung nodules which were inconsistent with pulmonary tuberculosis. A subsequent CT scan revealed erosive, lytic changes of the T11 and T12 vertebrae with compression of the T11 vertebra (Figure 1).

**Figure 2:**
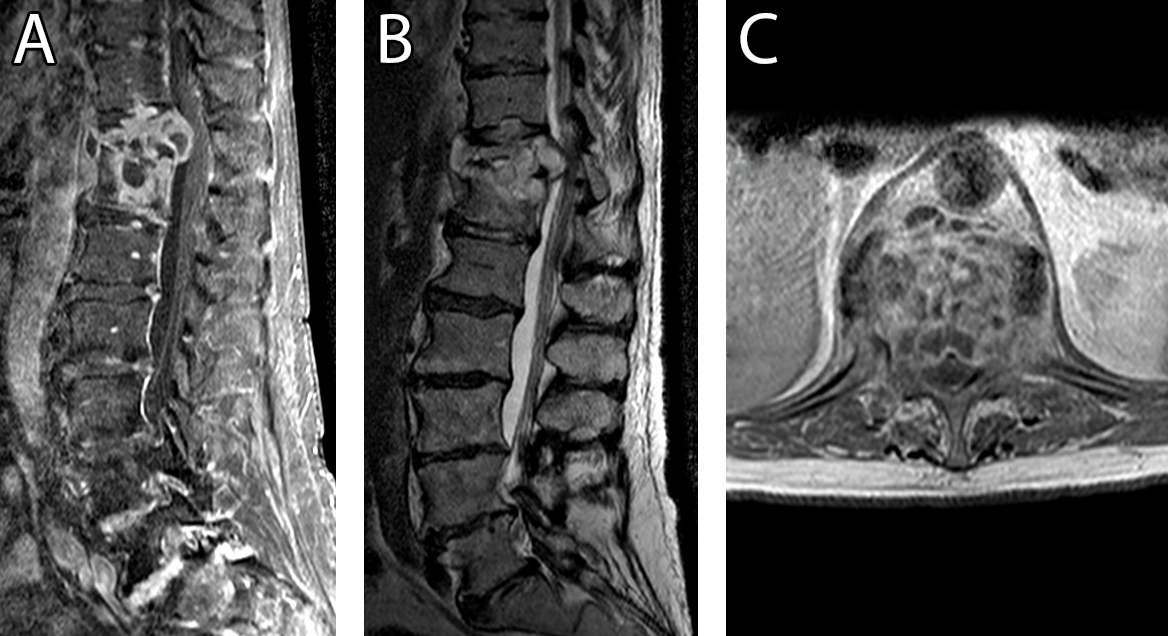
Preoperative MRI A) Preoperative sagittal T1-weighted MRI with contrast, B) sagittal T2-weighted MRI demonstrating T11-12 osteomyelitis with intervening discitis and paraspinal enhancement, and C) axial T1-weighted MRI with contrast demonstrating epidural phlegmon resulting in neural compression.

An MRI scan demonstrated T11-12 osteomyelitis with intervening discitis, paraspinal enhancement, and corresponding hyperintensity on a short tau inversion recovery (STIR) sequence consistent with an infectious process (Figure 2) [9]. A CT-guided needle aspiration yielded a negative gram stain and acid-fast bacillus (AFB) smear but grew *Mycobacterial tuberculosis *complex that was pansensitive to all therapies, except pyrazinamide on culture, a result characteristic of an *M. bovis *infection* *[10].

The patient began antimicrobial therapy that included rifabutin, ethambutol, isoniazid, and capreomycin. Capreomycin was discontinued due to renal insufficiency; cycloserine and moxifloxacin were ultimately added to the regimen in its place. Despite this aggressive antimicrobial therapy, the patient’s infection demonstrated radiographic evolution over the next two months, at which point he was evaluated by our institution. He had developed progressive kyphosis with intractable T11 and T12 radiculopathies due to nerve root compression, and surgical intervention was recommended. The patient underwent transthoracic T11-L2 vertebrectomies with decompression of the spinal cord and nerve roots as well as a T10-L1 instrumented fusion. After surgery, the patient was immobilized in a thoracolumbosacral orthosis (TLSO) brace and referred to physical therapy for 12 weeks with continued antimicrobial therapy consisting of rifabutin, ethambutol, isoniazid, cycloserine, and moxifloxacin.

**Figure 3:**
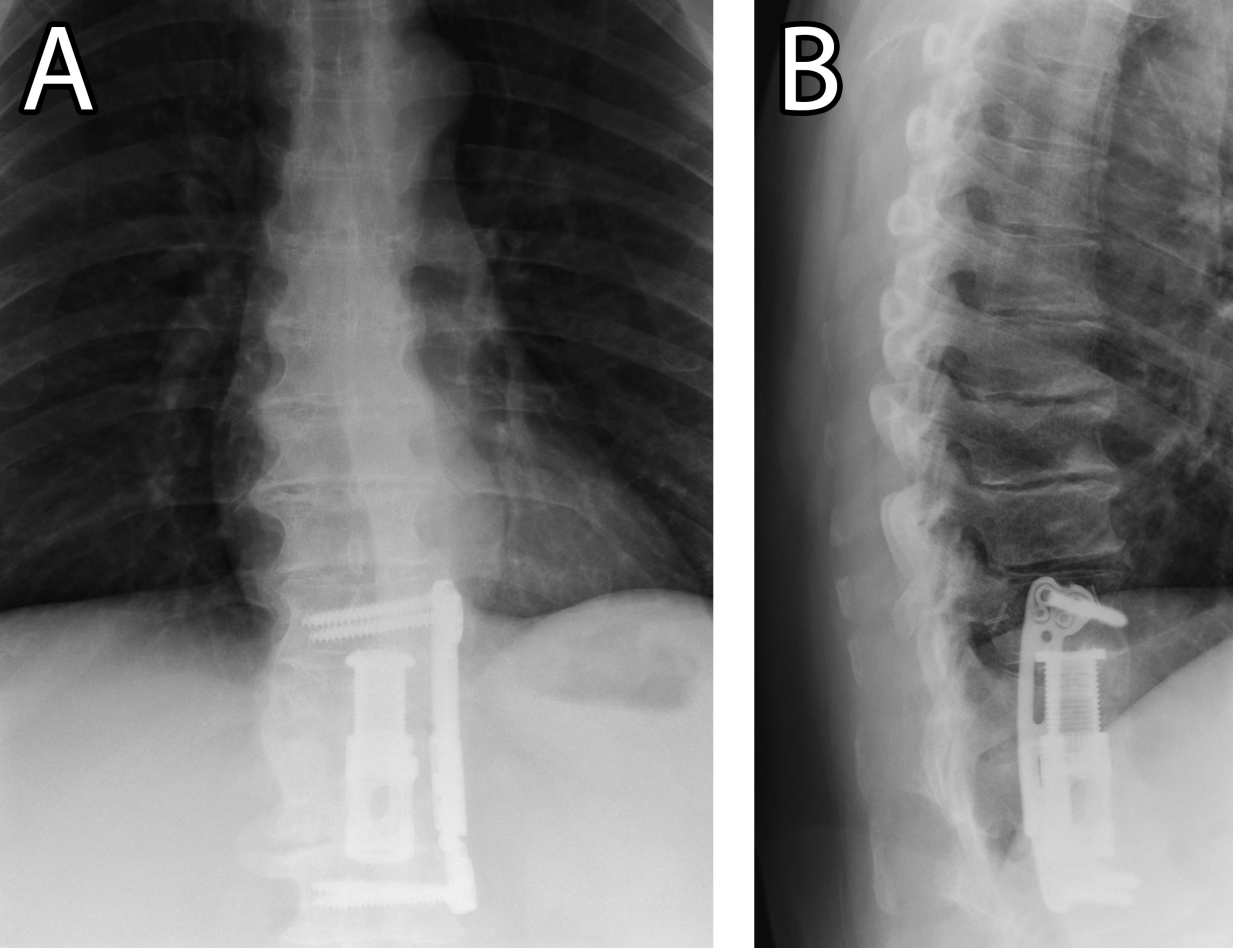
Postoperative lumbar X-rays A) Postoperative anteroposterior and B) lateral lumbar X-rays demonstrating the final spinal alignment, position of instrumentation, and resolution of infection one-year postoperatively.

The patient reported dramatic improvement in his pain and the ability to ambulate with a cane eight months postoperatively. Imaging studies 12 months postoperatively demonstrated continued remission of any infection and stable instrumentation (Figure 3). At the recommendation of our infectious disease service, his antimicrobial regimen was maintained and will likely be maintained for about one year in total.

## Discussion

In this case, we postulated that the intravesical BCG treatments had introduced an *M. bovis* infection, which had disseminated to cause vertebral osteomyelitis following the localized trauma of the second transurethral resection [5].

A review of the literature revealed 16 cases of *M. bovis *vertebral osteomyelitis secondary to intravesicular BCG therapy [5, 8, 11-24]. To appropriately evaluate the etiology of the cases, the review was restricted to the English language. All cases occurred in men between the ages of 58 and 94 with a time of onset ranging from two weeks to 12 years following BCG treatment. The most commonly affected vertebrae were T6-L5. As in our case, the axial back pain was the primary complaint. Weight loss [5, 8, 12, 20-21, 23-24] or lower extremity radiculopathies [8, 12, 14, 19-21] were secondary complaints in 9/16 cases. Antimicrobial TB therapy avoided surgical intervention in 9/16 cases [11, 13, 15-18, 20-22]. Isoniazid and rifampicin were the first-line agents of choice for *M. bovis*, frequently combined with ethambutol [5, 8, 13, 16-17, 20-23] and occasionally concomitant second-line agents [18, 21].

Potential trauma to the bladder endothelium (unrelated to BCG instillation) was described in 7/16 cases [13, 15-18, 22-23]. Such trauma included a biopsy or resection of the bladder [15-16, 22], pelvic radiation [17], radical cystoprostatectomy [13, 16], endovascular aneurysm repair of the abdominal aorta [18], and motor vehicle accident [23]. In the remaining nine cases, BCG instillation itself and the related instrumentation was considered to be the most likely source of endothelial trauma [5, 8, 11-12, 14, 19-21, 24].

Clinicians should be aware that traumatic intravesicular BCG therapy is not the sole mechanism of *M. bovis* spinal tuberculosis in adults. Alternative routes of entry include bovine contact and unpasteurized milk ingestion [25-27], adolescent BCG vaccination [28], intralesional BCG for non-bladder carcinoma [29], and incidental occupational exposure during BCG administration [30]. These cases have a wider age range (31-72 years old), frequently affect both sexes, and may involve cervical [30] or sacral [25] vertebrae.

The urinary tract is a major source of acute vertebral osteomyelitis [31-33]. Batson’s plexus may be responsible for the venous translocation of urinary tract infections to the internal vertebral venous column [34]. Others have previously speculated that intravesical BCG follows this hematogenous path when precipitating tuberculous spondylitis [5].

Despite the prevalence of genitourinary tract infections in women, vertebral osteomyelitis secondary to urinary tract infections most frequently occur in elderly men [34]. In fact, no cases of female vertebral osteomyelitis following intravesical BCG have been described in the English literature. Since bladder cancer is four times as common in men than women [35], a higher prevalence of case reports describing male patients is to be expected. However, male and female bladder cancer patients receive at least some form of intravesical BCG therapy at similar frequencies [36]. It is, therefore, curious that every known case of intravesical BCG-induced spondylitis occurred in a male.

This disproportion may be due to the presence of the prostatic venous plexus in men. In males, the vesical venous plexus may drain into the prostatic venous plexus, offering a direct path to the vertebral venous plexus. Females have no corresponding structure. Thus, urinary tract infections in females must take a much more circuitous route to the vertebral bodies via the internal iliac veins. These anatomic variances between genders may explain the disproportionately greater number of male cases of BCG osteomyelitis.

No formal American guidelines exist for managing vertebral osteomyelitis. As-directed antimicrobial therapy often resolves osteomyelitis without the need for surgery, initial treatment should be pathogen-directed [1]. Pyrazinamide-resistant tuberculosis, such as *M. bovis*, is most commonly treated with two months of isoniazid, rifampicin, and ethambutol followed by seven additional months of isoniazid and rifampicin. When TB suspicion is high, therapy should begin empirically after cultures have been obtained [10]. Pathologic deterioration despite antimicrobial therapy may require debridement and instrumentation may be necessary if the stability of the vertebral column is compromised [37]. Indications for surgical debridement, spinal decompression, and stabilization include the progression of infection despite antimicrobial therapy, spine deformity (e.g., kyphosis), pathologic fracture with intractable pain, and neurologic compromises, such as myelopathy and radiculopathy.

## Conclusions

A rare cause of vertebral osteomyelitis is *Mycobacterium bovis* after intravesical BCG therapy for transitional cell carcinoma of the bladder. In males, the vesical venous plexus can drain into the prostatic venous plexus and offer a direct path of hematogenous spread to the vertebral venous plexus. Despite aggressive antimicrobial therapy, *M. bovis* may demonstrate radiographic evolution with worsening neurological sequelae. Surgical debridement and vertebrectomies with instrumented fusion at the affected levels can help reduce the infection and improve clinical symptoms.
